# Geographic Distribution of Mental Health Problems Among Chinese College Students During the COVID-19 Pandemic: Nationwide, Web-Based Survey Study

**DOI:** 10.2196/23126

**Published:** 2021-01-29

**Authors:** Xiaoyan Wu, Shuman Tao, Yi Zhang, Shiyue Li, Le Ma, Yizhen Yu, Guilong Sun, Tingting Li, Fangbiao Tao

**Affiliations:** 1 Department of Maternal, Child and Adolescent Health School of Public Health Anhui Medical University Anhui China; 2 MOE Key Laboratory of Population Health Across Life Cycle Anhui China; 3 NHC Key Laboratory of Study on Abnormal Gametes and Reproductive Tract Anhui China; 4 Department of Nephrology The Second Hospital of Anhui Medical University Anhui China; 5 School of Health Sciences Wuhan University Wuhan China; 6 School of Public Health Xi’an Jiaotong University Health Science Center Xi'an China; 7 School of Public Health, Tongji Medical College Huazhong University of Science & Technology Wuhan China; 8 South-Central Minzu University Wuhan China

**Keywords:** COVID-19 pandemic, college students, mental health problems, geographic location

## Abstract

**Background:**

Since the COVID-19 outbreak was first reported, considerable attention has been drawn to mental health problems among college students.

**Objective:**

We aimed to estimate the prevalence of anxiety and depressive symptoms among college students in different geographical areas of China during the early stage of the COVID-19 outbreak.

**Methods:**

A nationwide cross-sectional survey was conducted among Chinese college students of 16 provinces or municipalities from February 4 to 12, 2020. A web-based survey was adopted to collect information from these college students, including demographics, perceived risk of infection, attitudes toward the epidemic and its control, and mental health status. Anxiety symptoms were assessed using the Generalized Anxiety Disorder scale, and depressive symptoms were assessed using the Patient Health Questionnaire. Chi-square test was used to compare the percentage of perceived risk of infection and attitude toward COVID-19 among college students in different geographic locations. Binary logistic models were used to identify associations between geographic locations and mental health problems after controlling for covariates.

**Results:**

A total of 11,787 participants were analyzed in this study (response rate: 79.7%). The prevalence of anxiety and depressive symptoms among college students was 17.8% (95% CI 17.1%-18.5%) and 25.9% (95% CI 25.1%-26.7%), respectively. After controlling for covariates, current residence area in Wuhan city was found to have a positive association with anxiety symptoms (odds ratio [OR] 1.37, 95% CI 1.11-1.68) and depressive symptoms (OR 1.32, 95% CI 1.09-1.59). Similarly, college location in Wuhan city was found to have a positive association with anxiety symptoms (OR 1.20, 95% CI 1.07-1.35) and depressive symptoms (OR 1.22, 95% CI 1.10-1.36). History of residence in or travel to Wuhan city in the past month was also positively associated with anxiety symptoms (OR 1.62, 95% CI 1.46-1.80) and depressive symptoms (OR 1.48, 95% CI 1.35-1.63). Furthermore, the perceived risk of COVID-19 was higher among students whose college location and current residence area were in Wuhan city, and it was positively associated with anxiety and depressive symptoms.

**Conclusions:**

During the COVID-19 pandemic, mental health problems among Chinese college students were widespread and geographically diverse. Our study results provide further insight for policymakers to develop targeted intervention strategies.

## Introduction

In December 2019, a new type of coronavirus named SARS-CoV-2 emerged in Wuhan, China [1]. This novel virus was found to cause a pneumonia-like respiratory infection called COVID-19; the disease rapidly spread all over China and turned into a pandemic, affecting most countries globally and putting the entire world on alert [2]. Because this is a novel disease, the preventive and treatment options were not clearly established at the beginning of the outbreak, imposing increased risk for mental health problems associated with COVID-19 [3]. The COVID-19 outbreak has also led to a major education crisis for children and adolescents. It is estimated that approximately 1.5 billion young people (ie, over 90% of all enrolled learners) worldwide were out of education [4], and more than 220 million children and adolescents in China were confined at home as a result of the COVID-19 outbreak [5]. Few studies have examined the effects of COVID-19 on young people, but studies on the general population have reported adverse mental health effects, including increased risks of anxiety, depression, general psychological distress, and posttraumatic stress disorder [6-8]. A recent study in China found that the general public’s vicarious traumatization scores were significantly higher than those of front-line nurses [9]. Health professionals and the general public were concerned with the potential adverse effects of the COVID-19 pandemic on the mental health of college students [10]. These concerns are consistent with earlier studies that have reported higher rates of mental health problems among college students following other disease outbreaks, such as SARS [11]. However, not much is known about the mental health effects of large-scale disease outbreaks on college students. Thus far, a few previous studies have allowed us to clarify the factors associated with mental health in a large population of college students after the COVID-19 outbreak.

The Chinese Lunar New Year is the largest annual event of mass travel worldwide [12]. In the context of the COVID-19 outbreak, tracking the migratory flow of college students in China is especially important because almost all of them were in the run-up to the annual Chunyun mass migration since the start of the winter holidays. Spatiotemporal patterns can help detect existing spatially clustered characteristics and high-risk areas, which can lead to a better understanding of the disease epidemic in the spatiotemporal dimension, thus providing reliable information for decision-making of disease prevention and control [13]. A compelling spatial analysis has identified hotspots within and outside of Hubei Province in China [14]. Wuhan is a city with many immigrants, and thousands of people left Wuhan to go to other cities and provinces before the COVID-19 lockdown came into effect. During this time, college students were also headed home for the winter holidays [15].

Therefore, we considered this specific population of college students in China to identify target locations for high risk of mental health problems. In this study, we aimed to estimate the prevalence of anxiety and depressive symptoms among college students in different geographical areas of China during the COVID-19 pandemic. It is essential to assess the spatial variability of mental health among Chinese college students during this pandemic, as it could provide useful insights to policymakers for targeted interventions.

## Methods

### Study Design and Participants

**Figure 1:**
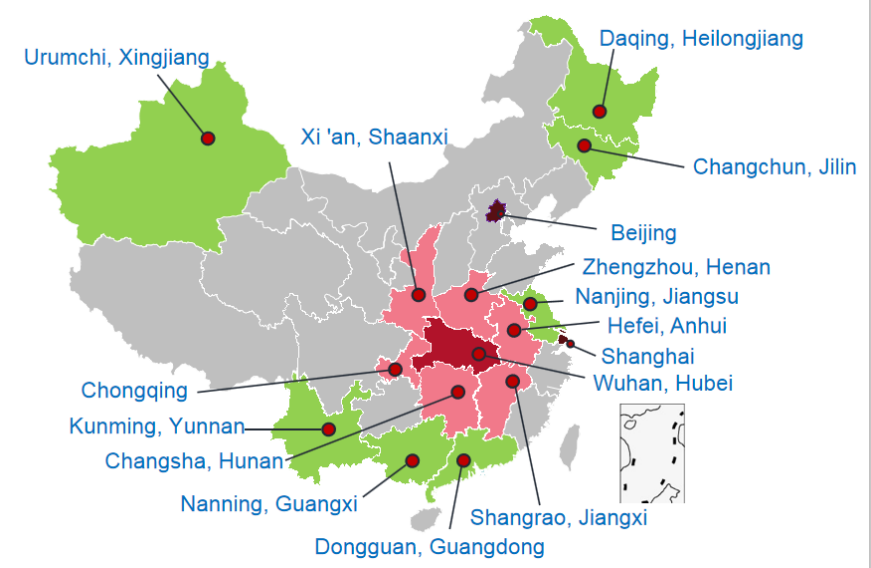
Distribution of study sample based on geographic locations of 16 provinces or municipalities in China.

This nationwide, cross-sectional, web-based survey was conducted from February 4 to February 12, 2020. A 2-stage sampling strategy was used. In the first stage, based on the geographic location and cooperation intention, we selected the following 16 provinces or municipalities: Wuhan city, neighboring provinces of Hubei (Henan, Anhui, Jiangxi, Hunan, Chongqing, and Shanxi), first-tier cities (Beijing and Shanghai), and other areas (Jiangsu, Guangdong, Guangxi, Yunnan, Xingjiang, Heilongjiang, and Jilin) (Figure 1). A total of 4 universities in Wuhan, Hubei Province, and 15 universities in other areas or municipalities were randomly selected for the analysis. In the second stage, 100-120 students of each grade (in general, 5 years for medical students and 4 years for nonmedical students) of a faculty were randomly selected from each university; these participants were invited to complete the web-based survey though the Wenjuanxing platform [16]. In total, 14,789 students were selected to participate in this study.

This study was approved by the Ethics Committee of Anhui Medical University. Electronic informed consent was obtained from all participants before the survey.

### Measurements

#### Geographic Location

Participants were asked to report their college location and current residence area. College locations were divided into the following 4 groups according to the geographic location: Wuhan city (the epicenter of the outbreak), neighboring provinces of Hubei, first-tier cities, and other provinces. Similarly, current residence areas were divided into the following 4 groups according to the geographic location: Wuhan city, other cities in Hubei Province, neighboring provinces of Hubei, and other provinces. Participants were also asked about their history of residence in or travel to Wuhan in the past month via a single question (yes, no).

To further understand the degree to which the COVID-19 pandemic had affected this student population, we divided the participants into the following groups according to the number of confirmed COVID-19 cases in their current residence area: <200, 200-600, 601-1000, and >1000. For this, we referred to the province-wise updates from the World Health Organization’s COVID-19 situation reports [17].

#### Perceived Risk of Infection and Attitude Toward COVID-19

Perceived risk of infection and attitudes toward COVID-19 were evaluated by the following questions: (1) How likely do you think you are at risk of COVID-19 infection? (2) How likely do you think are your family members at risk of COVID-19 infection? The responses to these questions were assigned the following scores: 1 (much less likely), 2 (less likely), 3 (more likely), and 4 (much more likely). Cronbach α was .876.

Participants’ worries about themselves and community members contracting the infection were evaluated by the following questions: (1) Do you worry about contracting the infection yourself? (2) Do you worry about infection among your community members? The responses were assigned the following scores: 1 (not at all), 2 (only a little), 3 (somewhat worry), and 4 (quite a lot). Cronbach α was .787.

Participants’ attitudes toward the COVID-19 epidemic and its control were evaluated by the following questions: (1) What is your attitude toward the COVID-19 epidemic? The responses to this question were assigned the following scores: 1 (very optimistic), 2 (somehow optimistic), 3 (somehow pessimistic), and 4 (very pessimistic). (2) Do you think the COVID-19 epidemic is hard to control at its current stage? The responses to this question were assigned the following scores: 1 (don’t agree), 2 (don’t agree or disagree), and 3 (agree). Cronbach α was .442.

#### Mental Health Problems

Participants’ anxiety symptoms were assessed by the Chinese version of the Generalized Anxiety Disorder (GAD-7) scale [18]. Participants were asked to rate the frequency of anxiety symptoms they experienced during the last 2 weeks. The GAD-7 scale contained 7 items that were scored on a 4-point scale, with scores ranging from 0 (not at all), 1 (several days), 2 (more than half the days), and 3 (nearly every day). The total score ranged from 0 to 21, with higher scores indicating higher GAD symptoms. The total score was categorized as mild, moderate, or severe anxiety based on cutoff scores of 5-9, 10-14, and 15-21, respectively.

Depressive symptoms were assessed using the Chinese version of the Patient Health Questionnaire (PHQ-9) [19], which is a 9-item questionnaire designed to screen for depressive symptoms. This questionnaire evaluated the frequency of depressive symptoms experienced by the participants in the last 2 weeks. Participants rated individual items on a 4-point scale, with 0 (not at all), 1 (several days), 2 (more than half the days), and 3 (nearly every day). The total score ranged from 0 to 27, with higher scores indicating higher levels of depression. The total score was categorized as mild, moderate, and severe depressive symptoms based on cutoff scores of 5-9, 10-14, and 15-27, respectively.

### Covariates

The following variables were included in the analysis as potential confounders: gender, age, and residence (urban and rural). Participants’ physical activity levels were assessed by the question “During the past 7 days, on how many days were you physically active for a total of at least 60 minutes per day? (Add all the time you spend in any kind of physical activity that increased your heart rate and made you breathe hard some of the time.)” [20]. The responses ranged from 0 to 7 days, and a high level of physical activity was defined as at least 3 days of exercise per week. Nighttime sleep duration was assessed by the following questions: “During the past week, when have you usually gone to bed?” “During the past week, when have you usually woken up?” [21]. Screen time was assessed using the following question: “How many hours per day did you spend on the computer (including playing video games or computer games or using a computer for something else) and watching TV/video programs during the past 7 days?” We categorized screen time as <2 hours/day (h/d), 2-4 h/d, and >4 h/d [22-24]. We also examined screen time spent viewing or obtaining COVID-19 information, and categorized it as <0.5 h/d, 0.5-1 h/d, and >1 h/d.

### Statistical Analyses

Categorical variables are presented as percentages (%) and frequencies (n). Continuous variables are presented as mean (SD) or median (IQR) values. Chi-square test was used to compare the percentages of perceived risk of infection and attitudes toward the COVID-19 pandemic among college students in different geographic locations. Binary logistic models were used to identify associations between geographic location and mental health problems after controlling for covariates. Variables that were potentially correlated with lifestyle during the COVID-19 pandemic and could cause a bias in the association with mental health, including gender, age, residence, nighttime sleep duration, physical activity, and screen time, were adjusted for in the regression models. We estimated the adjusted odds ratios (ORs) and 95% CIs of independent variables.

Data were analyzed using SPSS (version 23.0; IBM Corp). *P* values <.05 were considered statistically significant.

## Results

### Characteristics of the Study Sample

Survey responses from a total of 11,787 participants were analyzed in this study (response rate: 79.70%). The mean age of the study participants was 20.45 (SD 1.76) years, and 57.11% (6731/11,787) of all participants were female. Of all participants, 48.02% (5660/11,787) had a rural residence, 5.06% (597/11,787) reported they were currently residing in Wuhan, 41.46% (4887/11,787) attended a college located in Wuhan, and 26.52% (3126/11,787) had a history of residence in or travel to Wuhan in the past month. The prevalence of anxiety and depressive symptoms among the study participants was 17.80% (2098/11,787) and 25.90% (3053/11,787), respectively. Sample characteristics stratified by gender are shown in Table 1. The results of gender differences in risk perception are shown in the table in Multimedia Appendix 1. Our results showed that the high rate of perceived infection risk of participants themselves and their family members was more elevated among female participants than among male participants. Moreover, compared with male participants, female participants were more worried about themselves and their community members contracting the infection. The pessimistic attitude of female participants toward the COVID-19 epidemic was also higher than that observed among male participants. A larger proportion of female participants agreed that COVID-19 is difficult to control at the current stage. Prevalence of anxiety and depressive symptoms among college students with different demographic characteristics is shown in Multimedia Appendices 2 and 3, respectively.

**Table 1:** Characteristics of study participants by gender.

Characteristic		Total (N=11,787)		Male (n=5056)		Female (n=6731)		*t* test (*df*)		*C*hi-square *(df)*		*P* value	
Age, mean (SD)		20.45 (1.76)		20.44 (1.73)		20.46 (1.78)		-0.39 (11,785)		N/A^a^		.699	
Nighttime sleep duration (h/d), mean (SD)		9.57 (1.43)		9.46±1.55		9.65±1.32		-6.99 (11,785)		N/A		<.001	
**Grade, n (%)**								N/A		122.82 (4)		<.001	
	1		2930 (24.9)		1384 (27.4)		1546 (23)						
	2		2609 (22.1)		1201 (23.8)		1408 (20.9)						
	3		2667 (22.6)		1153 (22.8)		1514 (22.5)						
	4		2314 (19.6)		936 (18.5)		1378 (20.5)						
	5		1267 (10.7)		382 (7.6)		885 (13.1)						
**Residence, n (%)**								N/A		3.63 (1)		.06	
	Rural		5660 (48)		2479 (49)		3181 (47.3)						
	Urban		6127 (52)		2577 (51)		3550 (52.7)						
**Current residence area, n (%)**								N/A		41.53 (3)		<.001	
	Wuhan		597 (5.1)		235 (4.6)		362 (5.4)						
	Other cities in Hubei		2237 (19)		910 (18)		1327 (19.7)						
	Neighboring provinces of Hubei		2750 (23.3)		1323 (26.2)		1427 (21.2)						
	Other provinces		6203 (52.6)		2588 (51.2)		3615 (53.7)						
**College location, n (%)**								N/A		83.13 (3)		<.001	
	Wuhan		4887 (41.5)		1868 (36.9)		3019 (44.9)						
	Neighboring provinces of Hubei		2800 (23.8)		1336 (26.4)		1464 (21.8)						
	First-tier cities		900 (7.6)		434 (8.6)		466 (6.9)						
	Other provinces		3200 (27.1)		1418 (28)		1782 (26.5)						
**History of residence in or travel to Wuhan in the past month, n (%)**								N/A		10.51 (1)		.001	
	Yes		3126 (26.5)		1264 (25)		1862 (27.7)						
	No		8661 (73.5)		3792 (75)		4869 (72.3)						
**Physical activity, n (%)**								N/A		80.08 (1)		<.001	
	<3d/w		8334 (70.7)		3356 (66.4)		4978 (74)						
	≥3d/w		3453 (29.3)		1700 (33.6)		1753 (26)						
**Screen time, n (%)**								N/A		225.13 (2)		<.001	
	<2 h/d		2511 (21.3)		1395 (27.6)		1116 (16.6)						
	2-4 h/d		3706 (31.4)		1557 (30.8)		2149 (31.9)						
	>4 h/d		5570 (47.3)		2104 (41.6)		3466 (51.5)						
**Screen time spent viewing or obtaining COVID-19 information, n (%)**								N/A		110.80 (2)		<.001	
	<0.5 h/d		3231 (27.4)		1613 (31.9)		1618 (24)						
	0.5-1 h/d		4623 (39.2)		1965 (38.9)		2658 (39.5)						
	>1 h/d		3933 (33.4)		1478 (29.2)		2455 (36.5)						
**Anxiety symptoms, n (%)**								N/A		31.82 (1)		<.001	
	Yes		2098 (17.8)		784 (15.5)		1314 (19.5)						
	No		9689 (82.2)		4272 (84.5)		5417 (80.5)						
**Depressive symptoms, n (%)**								N/A		57.55 (1)		<.001	
	Yes		3053 (25.9)		1131 (22.4)		1922 (28.6)						
	No		8734 (74.1)		3925 (77.6)		4809 (71.4)						

^a^N/A: not applicable.

Participants whose current residence area and college were located in Wuhan had higher depressive and anxiety symptoms than those from other regions. Moreover, depressive and anxiety symptoms were more elevated in female participants than in male participants. Participants who had a history of residence in or travel to Wuhan in the past month also had higher depressive and anxiety symptoms than those without such history.

### Differences Between Current Residence Area or College Location and Perceived Risk of Infection and Attitude Toward COVID-19

Perceived risk of infection and attitudes toward COVID-19 for participants in different current residence areas and college location groups are shown in Multimedia Appendices 4 and 5, respectively. The percentage of high perceived infection risk for individuals was higher among participants who were currently residing in Wuhan and whose college area was located in Wuhan (both *P*<.001). Likewise, the percentage of high perceived infection risk for family members was higher among participants who were currently residing in Wuhan and whose college area was located in Wuhan (both *P*<.001). The percentage of participants who worried very much about themselves and their community members contracting the infection was higher among those whose college and current residence was located in Wuhan (both *P*<.001). The percentage of optimistic attitude toward COVID-19 was the lowest among college students whose current residence and college was located in Wuhan compared to those in the other groups. The highest proportion of college students currently residing in Wuhan agreed with the statement “COVID-19 is hard to control at its current stage” (*P*<.001); nevertheless, a median proportion of college students whose college area was located in Wuhan agreed with this statement (*P*<.001).

### Differences Between Perceived Risk of Infection and Attitude Toward COVID-19 and Mental Health

Overall, a strong gradient was observed between different levels of perceived infection risk for individuals themselves and for their family members with regard to the prevalence of anxiety and depression symptoms (Multimedia Appendices 6 and 7). Similarly, a strong gradient was also observed between different levels of worry about infection risk for individuals themselves and for their community members with regard to the prevalence of anxiety and depression symptoms (Multimedia Appendices 8 and 9). Furthermore, a gradient was observed between the different attitudes of college students toward the COVID-19 epidemic and its control and the prevalence of anxiety and depression symptoms (Multimedia Appendices 10 and 11).

Both anxiety and depression symptoms were more prevalent among college students who reported high perceived infection risk for themselves (*χ^2^*_2_=220.46 for anxiety, *χ^2^*_2_=131.79 for depression; both *P*<.001) and for their family members (*χ^2^*_2_=197.52 for anxiety, *χ^2^*_2_=136.06 for depression; both *P*<.001). The prevalence of anxiety and depression symptoms was the highest among college students who worried very much about themselves (*χ^2^*_3_=285.77 for anxiety, *χ^2^*_3_=186.10 for depression; both *P*<.001) and their community members (*χ^2^*_3_=178.46 for anxiety, *χ^2^*_3_=119.60 for depression; both *P*<.001) contracting the infection. The prevalence of anxiety and depression symptoms was the highest among college students who had a pessimistic attitude toward the COVID-19 epidemic (*χ^2^*_3_=444.61 for anxiety, *χ^2^*_3_=355.95 for depression; both *P*<.001) and its control (*χ^2^*_2_=154.02 for anxiety, *χ^2^*_2_=124.84 for depression; both *P*<.001).

### Associations Between Geographic Location and Mental Health

Tables 2 and 3 show the estimated ORs for the associations between geographic location and the prevalence of anxiety and depression among Chinese college students. Most associations were evident in both crude and adjusted models.

**Table 2:** Associations between geographic location and the prevalence of anxiety symptoms among college students in China.

Geographic location		Value, n (%)	Crude odds ratio (95% CI)	Adjusted odds ratio (95% CI)^a^
**Current residence area**				
	Wuhan	136 (22.8)	1.35 (1.10-1.65)^*^	1.37 (1.11-1.68)^*^
	Other cities in Hubei	368 (16.5)	0.90 (0.79-1.02)	0.92 (0.81-1.06)
	Neighboring provinces of Hubei	478 (17.4)	0.96 (0.85-1.08)	0.99 (0.88-1.12)
	Other provinces	1116 (18)	1.00	1.00
**College location**				
	Wuhan	967 (19.8)	1.18 (1.05-1.33)^*^	1.20 (1.07-1.35)^*^
	Neighboring provinces of Hubei	461 (16.5)	0.94 (0.82-1.08)	0.96 (0.84-1.10)
	First-tier cities	117 (13)	0.72 (0.58-0.89)^*^	0.72 (0.58-0.89)^*^
	Other provinces	553 (17.3)	1.00	1.00
**History of residence in or travel to Wuhan in the past month**				
	No	1363 (15.7)	1.00	1.00
	Yes	735 (23.5)	1.65 (1.49-1.82)^**^	1.62 (1.46-1.80)^**^
**Confirmed COVID-19 cases in current residence area**				
	<200	348 (16.8)	1.00	1.00
	200-600	712 (18.2)	1.10 (0.95-1.26)	1.09 (0.95-1.26)
	601-1000	513 (17.7)	1.07 (0.92-1.24)	1.10 (0.94-1.28)
	>1000	525 (18)	1.09 (0.94-1.26)	1.12 (0.96-1.31)

^a^Controlled for gender, age, residence, grade, nighttime sleep duration, physical activity, and screen time.

^*^P<.01.

^**^P<.001.

**Table 3:** Associations between geographic location and the prevalence of depressive symptoms among college students in China.

Geographic location		Value, n (%)	Crude odds ratio (95% CI)	Adjusted odds ratio (95% CI)^a^
**Current residence area**				
	Wuhan	187 (31.3)	1.35 (1.12-1.62)^*^	1.32 (1.09-1.59)^*^
	Other cities in Hubei	579 (25.9)	1.03 (0.92-1.15)	1.02 (0.91-1.15)
	Neighboring province of Hubei	718 (26.1)	1.04 (0.94-1.16)	1.08 (0.97-1.20)
	Other provinces	1569 (25.3)	1.00	1.00
**College location**				
	Wuhan	1393 (28.5)	1.26 (1.14-1.40)^**^	1.22 (1.10-1.36)^**^
	Neighboring provinces of Hubei	697 (24.9)	1.05 (0.93-1.18)	1.07 (0.95-1.21)
	First-tier cities	195 (21.7)	0.88 (0.73-1.05)	0.89 (0.75-1.07)
	Other provinces	768 (24­)	1.00	1.00
**History of residence in or travel to Wuhan in the past month**				
	No	2043 (23.6)	1.00	1.00
	Yes	1010 (32.3)	1.55 (1.41-1.69)^**^	1.48 (1.35-1.63)^**^
**Confirmed COVID-19 cases in current residence area**				
	<200	524 (25.4)	1.00	1.00
	200-600	1030 (26.3)	1.05 (0.93-1.87)	1.03 (0.91-1.17)
	601-1000	713 (24.7)	0.96 (0.85-1.10)	0.97 (0.85-1.11)
	>1000	786 (27)	1.09 (0.96-1.24)	1.06 (0.92-1.21)

^a^Controlled for gender, age, grade, residence, nighttime sleep duration, physical activity, and screen time.

^*^P<.01.

^**^P<.001.

Compared with the college students currently residing in other provinces, those currently residing in Wuhan city were significantly more likely to be at risk for symptoms of anxiety (crude OR 1.35, 95% CI 1.10-1.65, *P*<.01; adjusted OR 1.37, 95% CI 1.11-1.68, *P*<.01) and depression (crude OR 1.35, 95% CI 1.12-1.62, *P*<.01; adjusted OR 1.32, 95% CI 1.09-1.59, *P*<.01). Similarly, compared with the students whose colleges were located in other provinces, those whose colleges were located in Wuhan had a significantly increased risk of anxiety (crude OR=1.18, 95% CI 1.05-1.33, *P*<.01; adjusted OR 1.20, 95% CI 1.07-1.35, *P*<.01) and depression (crude OR 1.26, 95% CI 1.14-1.40, *P*<.001; adjusted OR 1.22, 95% CI 1.10-1.36, *P*<.001). Furthermore, students who had a history of residence in or travel to Wuhan in the past month were significantly more likely to be at risk for symptoms of anxiety and depression than those without such history (anxiety: crude OR 1.65, 95% CI 1.49-1.82, *P*<.001; adjusted OR 1.62, 95% CI 1.46-1.80, *P*<.001; depression: crude OR 1.55, 95% CI 1.41-1.69, *P*<.001; adjusted OR 1.48, 95% CI 1.35-1.63, *P*<.001). All associations were evident in both crude and adjusted models (Tables 2 and 3).

Nevertheless, participants’ currently living in areas with a median number (200-600, >1000) of confirmed COVID-19 cases were more likely to be at risk for both anxiety and depression symptoms compared with than were those currently living in areas with <200 confirmed cases; however, these associations were not statistically significant.

## Discussion

### Principal Findings

The COVID-19 crisis has caused significant public concern in China and around the world. Therefore, there is an urgent need to understand the psychological status of college students in different areas affected by the COVID-19 outbreak. To our knowledge, some studies have described the mental health status of Chinese college students during the COVID-19 epidemic [25,26], but these studies have not evaluated geographical location in detail. In this study, we adopted a web-based questionnaire to explore the association between mental health problems and geographic distribution. Our study provides important findings about the psychological well-being of college students across different geographical locations, which can help policymakers design targeted interventions in order to effectively improve the mental health status of these students.

We found that anxiety and depressive symptoms were positively associated with Wuhan-based geographic locations (ie, current residence area or college location) as well as history of residence in or travel to in Wuhan. Moreover, female students had a higher prevalence of anxiety and depression, which could be because women are considered to be more perceptual, emotional, relatively vulnerable to tension, and have a high incidence of depressive symptoms than men [27,28]. These findings implicate that intervention strategies should primarily focus on college students who studied, lived, or ever traveled to Wuhan city during the COVID-19 pandemic, so as to provide useful insights for policymakers for targeted intervention of COVID-19.

During the outbreak, college students were concerned about the possibility of being infected with COVID-19. In the present study, significant differences were observed between students’ current residence area or college location and perceived risk of infection and attitude toward COVID-19. Similarly, de Zwart [29] revealed the relatively high perceived threat for SARS. Public perception of health risk plays a key role in adopting these actions, in people’s feelings, and their daily habits [30]. Furthermore, our results revealed that the perceived risk of infection and attitudes toward COVID-19 were both associated with increased risks of anxiety and depressive symptoms. Similar to previous studies, increased worry about contracting infection and lower self-perceived health conditions were significantly associated with higher scores on the Self-Rating Anxiety Scale and Self-Rating Depression Scale [31]. Fear of the unknown increases anxiety levels among healthy individuals as well as those with preexisting mental health conditions [7]. As emotional responses will likely include extreme fear and uncertainty, this can further predict mental health consequences.

During the COVID-19 lockdown period, anxiety and depression among people substantially increased [8]. In the present study, 17.8% and 25.9% of respondents reported anxiety and depressive symptoms, respectively. One of the strengths of our study was that we used nationally representative data collected during the early stage of the COVID-19 outbreak in China and geographic location groups to explore mental health distribution. Currently, a large body of study has utilized web-based or mobile geographic information systems and mapping dashboards for tracking the COVID-19 epidemic [32,33]. The COVID-19 outbreak affected different locations such as Wuhan city or regions outside the Hubei Province, to varying levels [14]. A clear and comprehensive understanding of the effects of the COVID-19 epidemic on the mental health problems of college students residing and attending college across different geographic locations would effectively provide target prevention and control strategies.

We found that anxiety and depressive symptoms were positively associated with the current residence area and college locations based in Wuhan as well as with history of residence or travel to Wuhan. The evidence suggests that, in the early stages of the COVID-19 outbreak in Wuhan, rumors and misinformation were more prevalent in those areas and could cause anxiety and stress about the outbreak [34]. In particular, people who contracted the disease may be more vulnerable to the psychosocial effects than others [3]. Given that college students who were currently residing or attending college in Wuhan, as well as those who had a history of residence in or travel to Wuhan, likely had a higher risk of exposure to infection, there may be greater concern about infection. Moreover, college students residing in Wuhan during the outbreak may have experienced a shortage of personal protective equipment and had inadequate supplies, medical care, and medications during home confinement; all these factors would have likely increased the risks of mental health problems [3].

Our study mapped the effects of geographic location on spatial variability of mental health, and the findings would aid policymakers in developing targeted interventions. Recent studies among university studies during the COVID-19 pandemic have found similar results. For instance, Xin et al [35] reported that the prevalence of depression was 14.8%. Wang et al [36] demonstrated that the prevalence of anxiety and depressive symptoms was 7.7% and 12.2%, respectively. Another study conducted in Hubei Province among medical college students reported that 35.5% of the students experienced depression, and 22.1% experienced anxiety, and most students who were depressed or anxious had mild or moderate conditions [37]. Similar results were found in an American survey, wherein 71% of the participants reported increased stress and anxiety due to the COVID-19 outbreak [38]. The reason for these high levels of depression and anxiety could be attributed to the severity of the epidemic in the United States. These results show that the psychological condition of students during the epidemic cannot be ignored.

### Strengths and Limitations

This study evaluated the effects of COVID-19 on mental health among the Chinese youth. The investigation is representative of youth in China during the COVID-19 outbreak, and a relatively high response rate was achieved. The large national sample provided enough statistical power to examine the geographic location and mental health status of the participants. Our results provide further insight into developing targeted intervention strategies, including particular efforts undertaken by vulnerable populations [39,40]. Further research is required to study the effectiveness of prevention and control strategies, such as web-based mental health services [41], to promote mental health among college students during the COVID-19 epidemic.

However, this study has some potential limitations. First, this is a cross-sectional study, so it is difficult to elucidate causal relationships accurately. Additional longitudinal studies are necessary in the future. Nevertheless, we conducted follow-up examinations every 3 months for the study participants, thus enabling further clarification of the causal relationship. Second, despite the convenience of rapid assessment, our web-based survey may have potential respondent bias.

### Conclusions

Based on our findings, we can conclude that the geographic location of students’ current residence area or college location had a positive association with anxiety and depressive symptoms if based in Wuhan, compared with other areas. Moreover, a history of residence in or travel to Wuhan was also positively associated with anxiety symptoms and depressive symptoms. Thus, based on the geographic location of the college location and current residence area, college students in Wuhan city had a higher risk perception toward COVID-19.

## Appendix

Multimedia Appendix 1 Gender differences in risk perception.

Multimedia Appendix 2 Prevalence of anxiety symptom among college students with different demographic characteristics. Values are reported as n(%).

Multimedia Appendix 3 Prevalence of depression symptom among college students with different demographic characteristics. Values are reported as n(%).

Multimedia Appendix 4 Association between perceived risk of infection, attitude toward COVID-19, and college location. Values reported as n (%).

Multimedia Appendix 5 Association between perceived risk of infection, attitude toward COVID-19, and current residence area. Values reported as n (%).

Multimedia Appendix 6 Association of perceived infection risk for participants themselves with the prevalence of anxiety and depression symptoms.

Multimedia Appendix 7 Association of perceived infection risk for family members with the prevalence of anxiety and depression symptoms.

Multimedia Appendix 8 Association of worry about infection risk for participants themselves with the prevalence of anxiety and depression symptoms.

Multimedia Appendix 9 Association of worry about infection risk for community members with the prevalence of anxiety and depression symptoms.

Multimedia Appendix 10 Association of the attitude toward the COVID-19 epidemic with the prevalence of anxiety and depression symptoms.

Multimedia Appendix 11 Association of the attitude toward the control of COVID-19 with the prevalence of anxiety and depression symptoms.

## Abbreviations

GAD-7: 7-item Generalized Anxiety Disorder
OR: odds ratio
PHQ-9: 9-item Patient Health Questionnaire
