# Supplementary material for: Geographic Distribution of Mental Health Problems Among Chinese College Students During the COVID-19 Pandemic: Nationwide, Web-Based Survey Study
Source: J Med Internet Res. 2021 Jan 29;23(1):e23126. doi: 10.2196/23126 (PMC7850781; doi:10.2196/23126)
Supplement: Multimedia Appendix 1 [file jmir_v23i1e23126_app1.doc]

| Supplementary Table S1 The gender difference in risk perception | | | | | |
| --- | --- | --- | --- | --- | --- |
| Risk perception variables | | Male (n=5056), n(%) | Female (n=6731), n(%) | *χ*^2^value | *P* value |
| **How likely do you think you are at risk of COVID-19 infection?** | | | | 219.84 | <.001 |
|  | Much less likely | 2722 (53.8) | 2717 (40.4) |  |  |
|  | Less likely | 2094 (41.4) | 3647 (54.2) |  |  |
|  | More likely | 198 (3.9) | 330 (4.9) |  |  |
|  | Much more likely | 42 (0.8) | 37 (0.5) |  |  |
| **How likely do you think are your family members’ at risk of COVID-19 infection?** | | | | 229.49 | <.001 |
|  | Much less likely | 2702 (53.4) | 2663 (39.6) |  |  |
|  | Less likely | 2051 (40.6) | 3535 (52.5) |  |  |
|  | More likely | 257(5.1) | 481(7.1) |  |  |
|  | Much more likely | 46 (0.9) | 52 (0.8) |  |  |
| **Do you** **worry about contracting the infection yourself?** | | | | 385.86 | <.001 |
|  | Not at all | 1392(27.5) | 894(13.3) |  |  |
|  | Only a little | 2294(45.4) | 3454(51.3) |  |  |
|  | Somewhat worry | 675(13.4) | 1162(17.3) |  |  |
|  | Quite a lot | 695(13.7) | 1221(18.1) |  |  |
| **Do you worry about infection among your community members?** | | | | 343.63 | <.001 |
|  | Not at all | 720(14.2) | 365(5.4) |  |  |
|  | Only a little | 2636(52.1) | 3338(49.6) |  |  |
|  | Somewhat worry | 987(19.5) | 1675(24.9) |  |  |
|  | Quite a lot | 713(14.1) | 1353(20.1) |  |  |
| **What is your attitude toward the COVID-19 epidemic?** | | | | 224.58 | <.001 |
|  | Very optimistic | 1277(25.3) | 985(14.6) |  |  |
|  | Somehow optimistic | 2710(53.6) | 4170(62.0) |  |  |
|  | Somehow pessimistic | 940(18.6) | 1454(21.6) |  |  |
|  | Very pessimistic | 129(2.6) | 122(1.8) |  |  |
| **Do you think the COVID-19 epidemic is hard to control at current stage?** | | | | 91.30 | <.001 |
|  | Don’t agree | 1952(38.6) | 2070(30.8) |  |  |
|  | Don’t agree or disagree | 1685(33.3) | 2718(40.4) |  |  |
|  | Agree | 1419(28.1) | 1943(28.8) |  |  |
