# Supplementary material for: Geographic Distribution of Mental Health Problems Among Chinese College Students During the COVID-19 Pandemic: Nationwide, Web-Based Survey Study
Source: J Med Internet Res. 2021 Jan 29;23(1):e23126. doi: 10.2196/23126 (PMC7850781; doi:10.2196/23126)
Supplement: Multimedia Appendix 2 [file jmir_v23i1e23126_app2.docx]

| Supplementary Table S2 Prevalence of anxiety symptom among college students with different demographic characteristics | | | | | | | | |
| --- | --- | --- | --- | --- | --- | --- | --- | --- |
| Characteristic | | Total  (N=11787) | Anxiety symptom (N=2098), n(％) | | | | *χ*^2^ value | *P* value |
|  |  |  | Overall | Mild | Moderate | Severe |  |  |
| **Gender** | | | | | | | 45.83 | <.001 |
|  | Male | 5056(42.9) | 784(15.5) | 509(10.1) | 204(4.0) | 71(1.4) |  |  |
|  | Female | 6731(57.1) | 1314(19.5) | 956(14.2) | 258(3.8) | 100(1.5) |  |  |
| **Grade** |  |  |  |  |  |  | 47.17 | <.001 |
|  | 1 | 2930(24.9) | 417(14.2) | 311(10.6) | 73(2.5) | 33(1.1) |  |  |
|  | 2 | 2609(22.1) | 499(19.1) | 333(12.8) | 123(4.7) | 43(1.6) |  |  |
|  | 3 | 2667(22.6) | 525(19.7) | 370(13.9) | 112(4.2) | 43(1.6) |  |  |
|  | 4 | 2314(19.6) | 433(18.7) | 292(12.6) | 101(4.4) | 40(1.7) |  |  |
|  | 5 | 1267(10.7) | 224(17.7) | 159(12.5) | 53(4.2) | 12(0.9) |  |  |
| **Residence** | | | | | | | 3.63 | .304 |
|  | Rural | 5660(48.0) | 984(17.4) | 690(12.2) | 223(3.9) | 71(1.3) |  |  |
|  | Urban | 6127(52.0) | 1114(18.2) | 775(12.6) | 239(3.9) | 100(1.6) |  |  |
| **Current residence area** | | | | | | | 39.42 | <.001 |
|  | Wuhan | 597(5.1) | 136(22.8) | 86(14.4) | 27(4.5) | 23(3.9) |  |  |
|  | Other cities in Hubei | 2237(19.0) | 368(16.4) | 278(12.4) | 65(2.9) | 25(1.1) |  |  |
|  | Neighboring provinces of Hubei | 2750(23.3) | 478(17.4) | 322(11.7) | 120(4.4) | 36(1.3) |  |  |
|  | Other provinces | 6203(43.5) | 1116(18.0) | 779(12.6) | 250(4.0) | 87(1.4) |  |  |
| **College location** | | | | | | | 47.04 | <.001 |
|  | Wuhan | 4887(41.5) | 967(19.8) | 690(14.1) | 184(3.8) | 93(1.9) |  |  |
|  | Neighbour province of Hubei | 2800(23.8) | 461(16.5) | 323(11.5) | 109(3.9) | 29(1.0) |  |  |
|  | First-tier city | 900(7.6) | 117(13.0) | 81(9.0) | 25(2.8) | 11(1.2) |  |  |
|  | Other provinces | 3200(27.1) | 553(17.3) | 371(11.6) | 144(4.5) | 38(1.2) |  |  |
| **History of residence in or travel to Wuhan in the past month** | | | | | | | 109.47 | <.001 |
|  | Yes | 3126(26.5) | 735(23.5) | 496(15.9) | 158(5.1) | 81(2.6) |  |  |
|  | No | 8661(73.5) | 1363(15.7) | 969(11.2) | 304(3.5) | 90(1.0) |  |  |
| **Screen time** | | | | | | | 113.15 | <.001 |
|  | ＞4 h | 5570(47.3) | 1149(20.6) | 795(14.3) | 241(4.3) | 113(2.0) |  |  |
|  | 2-4 h | 3706(31.4) | 506(13.7) | 396(10.7) | 84(2.3) | 26(0.7) |  |  |
|  | ≤2 h | 2511(21.3) | 443(17.6) | 274(10.9) | 137(5.5) | 32(1.3) |  |  |
| **Physical activity** | | | | | | | 50.60 | <.001 |
|  | ≥3d | 3453(29.3) | 482(14.0) | 338(9.8) | 111(3.2) | 33(1.0) |  |  |
|  | ＜3d | 8334(70.7) | 1616(19.4) | 1127(13.5） | 351(4.2) | 138(1.7) |  |  |
