# Supplementary material for: Geographic Distribution of Mental Health Problems Among Chinese College Students During the COVID-19 Pandemic: Nationwide, Web-Based Survey Study
Source: J Med Internet Res. 2021 Jan 29;23(1):e23126. doi: 10.2196/23126 (PMC7850781; doi:10.2196/23126)
Supplement: Multimedia Appendix 3 [file jmir_v23i1e23126_app3.docx]

| Supplementary Table S3 Prevalence of depression symptom among college students with different demographic characteristics | | | | | | | | |
| --- | --- | --- | --- | --- | --- | --- | --- | --- |
| Characteristic | | Total (N=11787) | Depression symptom (N=3053), n(％) | | | | *χ*^2^ value | *P* value |
|  |  |  | Overall | Mild | Moderate | Severe |  |  |
| **Gender** | | | | | | | 77.07 | <.01 |
|  | Male | 5056(42.9) | 1131(22.4) | 709(14.0) | 208(4.1) | 214(4.3) |  |  |
|  | Female | 6731(57.1) | 1922(28.5) | 1313(19.5) | 361(5.4) | 248(3.7) |  |  |
| **Grade** |  |  |  |  |  |  | 21.68 | .041 |
|  | 1 | 2930(24.9) | 708(24.2) | 484(16.5) | 134(4.6) | 90(3.1) |  |  |
|  | 2 | 2609(22.1) | 707(27.1) | 453(17.4) | 137(5.3) | 117(4.5) |  |  |
|  | 3 | 2667(22.6) | 724(27.1) | 481(18.0) | 123(4.6) | 120(4.5) |  |  |
|  | 4 | 2314(19.6) | 619(26.8) | 407(17.6) | 117(5.1) | 95(4.1) |  |  |
|  | 5 | 1267(10.7) | 295(23.2) | 197(15.5) | 58(4.6) | 40(3.2) |  |  |
| **Residence** | | | | | | | 6.45 | .092 |
|  | Rural | 5660(48.0) | 1431(25.3) | 942(16.6) | 286(5.1) | 203(3.6) |  |  |
|  | Urban | 6127(52.0) | 1622(26.5) | 1080(17.6) | 283(4.6) | 259(4.2) |  |  |
| **Current residence area** | | | | | | | 20.58 | .015 |
|  | Wuhan | 597(5.1) | 187(31.3) | 114(19.1) | 38(6.4) | 35(5.9) |  |  |
|  | Other cities in Hubei | 2237(19.0) | 579(25.9) | 409(18.3) | 97(4.3) | 73(3.3) |  |  |
|  | Neighboring provinces of Hubei | 2750(23.3) | 718(26.1) | 481(17.5) | 134(4.9) | 103(3.7) |  |  |
|  | Other provinces | 6203(43.5) | 1569(25.3) | 1018(16.4) | 300(4.8) | 251(4.0) |  |  |
| **College location** | | | | | | | 44.66 | <.001 |
|  | Wuhan | 4887(41.5) | 1393(28.5) | 924(18.9) | 255(5.2) | 214(4.4) |  |  |
|  | Neighboring provinces of Hubei | 2800(23.8) | 697(24.9) | 480(17.1) | 136(4.9) | 81(2.9) |  |  |
|  | First-tier cities | 900(7.6) | 195(21.7) | 132(14.7) | 34(3.8) | 29(3.2) |  |  |
|  | Other provinces | 3200(27.1) | 768(24.0) | 486(15.2) | 144(4.5) | 138(4.3) |  |  |
| **History of residence in or travel to Wuhan in the past month** | | | | | | | 105.53 | <.001 |
|  | Yes | 3126(26.5) | 1010(32.3) | 640(20.5) | 184(5.9) | 186(6.0) |  |  |
|  | No | 8661(73.5) | 2043(23.6) | 1382(16.0) | 385(4.4) | 276(3.2) |  |  |
| **Screen time** | | | | | | | 195.87 | <.001 |
|  | ＞4 h | 5570(47.3) | 1741(31.3) | 1126(20.2) | 349(6.3) | 266(4.8) |  |  |
|  | 2-4 h | 3706(31.4) | 756(20.4) | 560(15.1) | 119(3.2) | 77(2.1) |  |  |
|  | ≤2 h | 2511(21.3) | 556(22.1) | 336(13.4) | 101(4.0) | 119(4.7) |  |  |
| **Physical activity** | | | | | | | 94.99 | <.001 |
|  | ≥3d | 3453(29.3) | 695(20.1) | 491(14.2) | 99(2.9) | 105(3.0) |  |  |
|  | ＜3d | 8334(70.7) | 2358(28.3) | 1531(18.4） | 470(5.6) | 357(4.3) |  |  |
