# Supplementary material for: Geographic Distribution of Mental Health Problems Among Chinese College Students During the COVID-19 Pandemic: Nationwide, Web-Based Survey Study
Source: J Med Internet Res. 2021 Jan 29;23(1):e23126. doi: 10.2196/23126 (PMC7850781; doi:10.2196/23126)
Supplement: Multimedia Appendix 4 [file jmir_v23i1e23126_app4.docx]

| Supplementary Table S4 Association between perceived risk of infection, attitude toward COVID-19, and college location | | | | | | | |
| --- | --- | --- | --- | --- | --- | --- | --- |
| Risk perception variables | | Total  (N=11787) | College location | | | | *P* value |
|  |  |  | Wuhan | Neighboring provinces of Hubei | First-tier cities | Other provinces |  |
| **Perceived infection risk of him/herself** | | | | | | | <.001 |
|  | Much less likely | 5439(46.1) | 2289(46.8) | 1246(44.5) | 458(50.9) | 1446(45.2) |  |
|  | Less likely | 5741(48.7) | 2305(47.2) | 1433(51.2) | 415(46.1) | 1588(49.6) |  |
|  | More likely and much more likely | 607(5.1) | 293(6.0) | 121(4.3) | 27(3.0) | 166(5.2) |  |
| **Perceived infection risk of family members** | | | | | | | <.001 |
|  | Much less likely | 5365(45.5) | 2388(48.9) | 1184(42.3) | 438(48.7) | 1355(42.3) |  |
|  | Less likely | 5586(47.4) | 2122(43.4) | 1446(51.6) | 418(46.4) | 1600(50.0) |  |
|  | More likely and much more likely | 836(7.1) | 377(7.7) | 170(6.1) | 44(4.9) | 245(7.7) |  |
| **Worry about infection of her/himself** | | | | | | | <.001 |
|  | Not at all | 2286(19.4) | 949(19.4) | 570(20.4) | 201(22.3) | 566(17.7) |  |
|  | Only a little | 5748(48.8) | 2262(46.3) | 1490(53.2) | 437(48.6) | 1559(48.7) |  |
|  | Somewhat worry | 1837(15.6) | 784(16.0) | 409(14.6) | 149(16.6) | 495(15.5) |  |
|  | Quite a lot | 1916(16.3) | 892(18.3) | 331(11.8) | 113(12.6) | 580(18.1) |  |
| **Worry about infection of community members** | | | | | | | <.001 |
|  | Not at all | 1085(9.2) | 447(9.1) | 254(9.1) | 92(10.2) | 292(9.1) |  |
|  | Only a little | 5974(50.7) | 2366(48.4) | 1541(55.0) | 463(51.4) | 1604(50.1) |  |
|  | Somewhat worry | 2662(22.6) | 1149(23.5) | 587(21.0) | 207(23.0) | 719(22.5) |  |
|  | Quite a lot | 2066(17.5) | 925(18.9) | 418(14.9) | 138(15.3) | 585(18.3) |  |
| **Attitude toward COVID-19 epidemic** | | | | | | | <.001 |
|  | Very [optimistic](C:/Program%20Files%20(x86)/Youdao/Dict/8.9.0.0/resultui/html/index.html#/javascript:;) | 2262(19.2) | 891(18.2) | 501(17.9) | 164(18.2) | 706(22.1) |  |
|  | Somehow optimistic | 6880(58.4) | 2813(57.6) | 1704(60.9) | 550(61.1) | 1813(56.7) |  |
|  | Somehow pessimistic | 2394(20.3) | 1076(22.0) | 553(19.8) | 174(19.3) | 591(18.5) |  |
|  | Very pessimistic | 251(2.1) | 107(2.2) | 42(1.5) | 12(1.3) | 90(2.8) |  |
| **COVID-19 is hard to control at current stage** | | | | | | | <.001 |
|  | Don’t agree | 4022(34.1) | 1508(30.9) | 1113(39.8) | 318(35.3) | 1083(33.8) |  |
|  | Don’t agree or disagree | 4403(37.4) | 2020(41.3) | 949(33.9) | 324(36.0) | 1110(34.7) |  |
|  | Agree | 3362(28.5) | 1359(27.8) | 738(26.4) | 258(28.7) | 1007(31.5) |  |
