# Supplementary material for: Geographic Distribution of Mental Health Problems Among Chinese College Students During the COVID-19 Pandemic: Nationwide, Web-Based Survey Study
Source: J Med Internet Res. 2021 Jan 29;23(1):e23126. doi: 10.2196/23126 (PMC7850781; doi:10.2196/23126)
Supplement: Multimedia Appendix 5 [file jmir_v23i1e23126_app5.docx]

| Supplementary Table S5 Association between perceived risk of infection, attitude toward COVID-19, and current residence area | | | | | | | |
| --- | --- | --- | --- | --- | --- | --- | --- |
| Risk perception variables | | Total (N=11787) | Current residence area | | | | *P* value |
|  |  |  | Wuhan | Other cities in Hubei | Neighboring provinces of Hubei | Other provinces |  |
| **Perceived infection risk of him/herself** | | | | | | | <.001 |
|  | Much less likely | 5439 (46.1) | 233(39.0) | 1044(46.7) | 1287(46.8) | 2875(46.3) |  |
|  | Less likely | 5741(48.7) | 296(49.6) | 1086(48.5) | 1350(49.1) | 3009(48.5) |  |
|  | More likely and much more likely | 607(5.1) | 68(11.4) | 107(4.8) | 113(4.1) | 319(5.2) |  |
| **Perceived infection risk of family members** | | | | | | | <.001 |
|  | Much less likely | 5365(45.5) | 225(37.7) | 1065(47.6) | 1232(44.8) | 2843(45.8) |  |
|  | Less likely | 5586(47.4) | 287(48.1) | 1016(45.4) | 1360(49.5) | 2923(47.1) |  |
|  | More likely and much more likely | 836(7.1) | 85(14.3) | 156(7.0) | 158(5.7) | 437(7.1) |  |
| **Worry about infection of her/himself** | | | | | | | <.001 |
|  | Not at all | 2286(19.4) | 118(19.8) | 392(17.5) | 560(20.4) | 1216(19.6) |  |
|  | Only a little | 5748(48.8) | 266(44.6) | 1057(47.3) | 1416(51.5) | 3009(48.5) |  |
|  | Somewhat worry | 1837(15.6) | 85(14.2) | 357(16.0) | 428(15.6) | 967(15.6) |  |
|  | Quite a lot | 1916(16.3) | 128(21.4) | 431(19.3) | 346(12.6) | 1011(16.3) |  |
| **Worry about infection of community members** | | | | | | | <.001 |
|  | Not at all | 1085(9.2) | 52 (8.7) | 163(7.3) | 246(8.9) | 624(10.1) |  |
|  | Only a little | 5974(50.7) | 258(43.2) | 1085(48.5) | 1488(54.1) | 3143(50.7) |  |
|  | Somewhat worry | 2662(22.6) | 146(24.5) | 542(24.2) | 606(22.0) | 1368(22.1) |  |
|  | Quite a lot | 2066(17.5) | 141(23.6) | 447(20.0) | 410(14.9) | 1068(17.2) |  |
| **Attitude toward COVID-19 epidemic** | | | | | | | <.001 |
|  | Very [optimistic](C:/Program%20Files%20(x86)/Youdao/Dict/8.9.0.0/resultui/html/index.html#/javascript:;) | 2262(19.2) | 102(17.1) | 421(18.8) | 487(17.7) | 1252(20.2) |  |
|  | Somehow optimistic | 6880(58.4) | 312(52.3) | 1321(59.1) | 1676(60.9) | 3571(57.6) |  |
|  | Somehow pessimistic | 2394(20.3) | 159(26.6) | 454(20.3) | 536(19.5) | 1245(20.1) |  |
|  | Very pessimistic | 251(2.1) | 24(4.0) | 41(1.8) | 51(1.9) | 135(2.2) |  |
| **COVID-19 is hard to control at its current stage** | | | | | | | <.001 |
|  | Don’t agree | 4022(34.1) | 152(25.5) | 655(29.3) | 1085(39.5) | 2130(34.3) |  |
|  | Don’t agree or disagree | 4403(37.4) | 246(41.2) | 959(42.9) | 953(34.7) | 2245(36.2) |  |
|  | Agree | 3362(28.5) | 199(33.3) | 623(27.8) | 712(25.9) | 1828(29.5) |  |
