# Supplementary figures and images for: Geographic Distribution of Mental Health Problems Among Chinese College Students During the COVID-19 Pandemic: Nationwide, Web-Based Survey Study
Source: J Med Internet Res. 2021 Jan 29;23(1):e23126. doi: 10.2196/23126 (PMC7850781; doi:10.2196/23126)

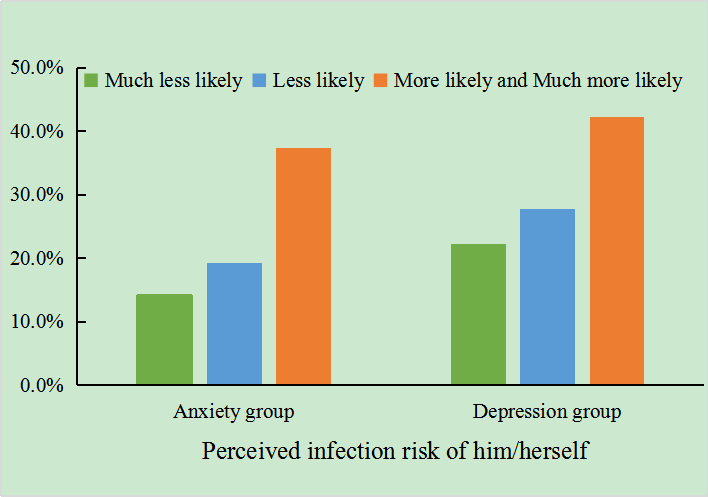

Supplement: Multimedia Appendix 6 [file jmir_v23i1e23126_app6.png]

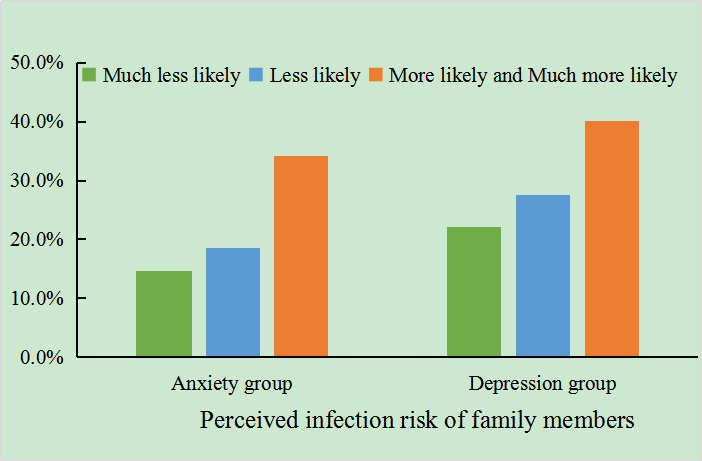

Supplement: Multimedia Appendix 7 [file jmir_v23i1e23126_app7.png]

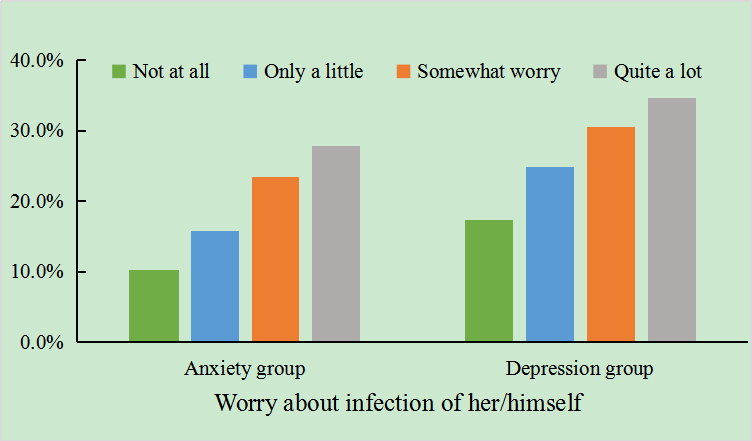

Supplement: Multimedia Appendix 8 [file jmir_v23i1e23126_app8.png]

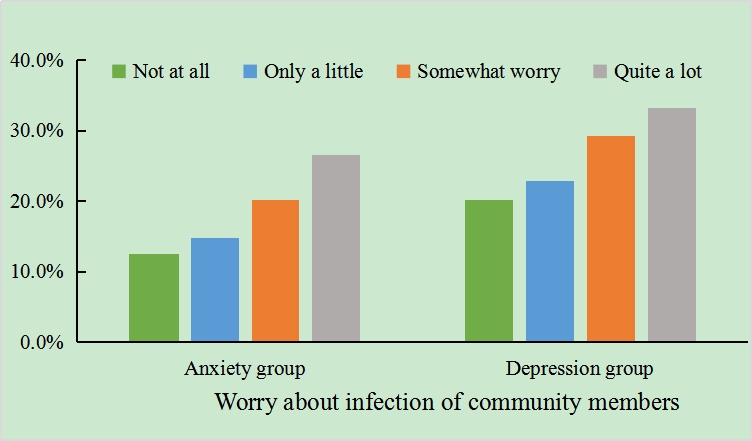

Supplement: Multimedia Appendix 9 [file jmir_v23i1e23126_app9.png]

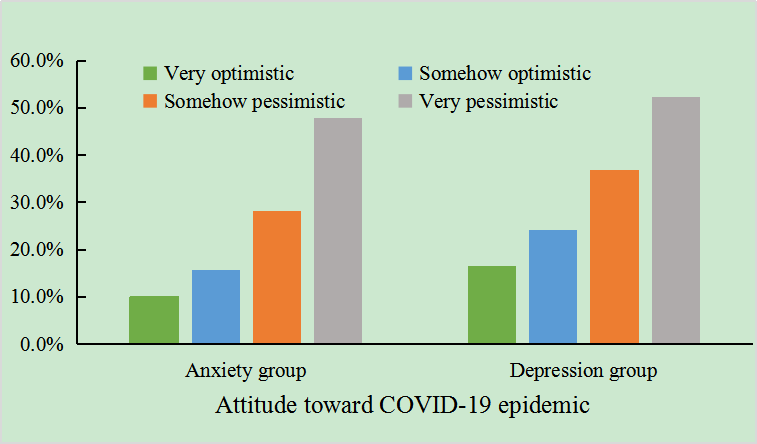

Supplement: Multimedia Appendix 10 [file jmir_v23i1e23126_app10.png]

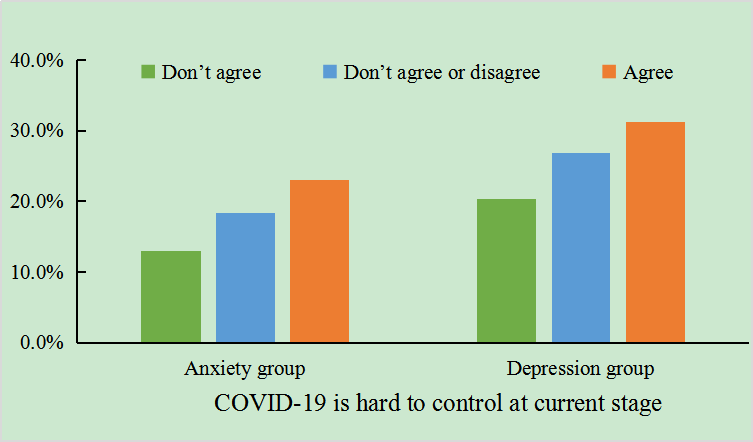

Supplement: Multimedia Appendix 11 [file jmir_v23i1e23126_app11.png]
